# Transmission and Characterization of Creutzfeldt–Jakob Disease and Chronic Wasting Disease in the North American Deer Mouse

**DOI:** 10.3390/v17040576

**Published:** 2025-04-16

**Authors:** Jennifer Myskiw, Lise Lamoureux, Kathy Frost, Rebecca Fox, Jessy A. Slota, Gordon Mitchell, Ben A. Bailey-Elkin, Stephanie A. Booth

**Affiliations:** 1 Mycobacteriology, Vector-Borne and Prion Diseases Division, National Microbiology Laboratory, Public Health Agency of Canada, Winnipeg, MB R3E 3R2, Canada; jennifer.myskiw@phac-aspc.gc.ca (J.M.); lise.lamoureux@phac-aspc.gc.ca (L.L.); kathy.frost@phac-aspc.gc.ca (K.F.); rebecca.fox@phac-aspc.gc.ca (R.F.); jessy.slota@phac-aspc.gc.ca (J.A.S.); ben.bailey-elkin@phac-aspc.gc.ca (B.A.B.-E.); 2 National and WOAH Reference Laboratory for Scrapie and CWD, Canadian Food Inspection Agency, Ottawa, ON K2H 8P9, Canada; gordon.mitchell@inspection.gc.ca; 3 Department of Medical Microbiology and Infectious Diseases, Faculty of Health Sciences, University of Manitoba, Winnipeg, MB R3T 2N2, Canada

**Keywords:** prion diseases, Creutzfeldt-Jakob disease, chronic wasting disease, neurodegenerative diseases

## Abstract

Prion transmission into rodents is essential for understanding prion strains. However, it is often limited by a “species barrier” that makes transmission challenging and complicates the study of animal and human prion diseases. Here, we report that North American deer mice (*Peromyscus maniculatus*) are susceptible to infection with both human sporadic Creutzfeldt–Jakob disease (sCJD) and chronic wasting disease (CWD). Experimental transmission of both sCJD and CWD in deer mice resulted in 100% attack rates, albeit with differing incubation times, with CWD-inoculated mice taking nearly three times longer than sCJD-inoculated mice to succumb. We observed distinct patterns of spongiform vacuolation and prion-protein deposition in the brain, as well as distinct protein-glycosylation profiles and seeding kinetics in RT-QuIC for each strain. Adaptation on the second passage led to reduced incubation periods and marked strain-specific pathology, as seen predominantly in the cortex in sCJD and the thalamus in CWD. Notably, primary transmission of CWD resulted in infrequent vacuoles and widespread punctate deposits of prion protein in the brain, while diffuse staining and remarkable vacuolation of the thalamus were seen on passage. Prion seeding kinetics for sCJD and CWD were indistinguishable in the second passage; however, the distinct glycosylation patterns seen on immunoblot of the prion protein were maintained. Adaptation also resulted in extraneural dissemination of prion seeding activity distinct to CWD infection. Overall, the ability to transmit both CWD and sCJD to this model, resulting in clear differences in incubation period, biochemical properties, clinical signs, pathology and seeding kinetics, indicates that the model has the potential for use as a tool to investigate atypical cases of sCJD that may indicate CWD spillover to humans.

## 1. Introduction

Transmissible spongiform encephalopathies (TSEs) represent a group of rare and invariably fatal neurodegenerative disorders that afflict humans and animals. Characterized by the accumulation of abnormal prion proteins in the central nervous system, TSEs pose a challenge to public health. Templated misfolding of host-encoded cellular prion protein (PrP^C^) into an infectious disease-associated isoform (PrP^Sc^) causes the distinctive protein deposits and spongiform changes in affected brain tissue. Infectious proteins spread from cell to cell, with prion replication occurring during a long, asymptomatic preclinical phase [1]. Neurotoxicity ultimately leads to neuronal loss and rapid neurodegenerative decline and death. The unique mode of transmission is driven by protein conformation rather than by nucleic acids, in contrast to viruses. Despite this, prions exist as different strains with unique pathological and clinical properties encoded within their primary structures. The primary sequence of the host prion protein influences prion strains’ transmission between species [2]. Primary-structure mismatches influence propagation kinetics and can prolong incubation periods and prevent infected animals from developing disease. However, passaging frequently results in the formation of a strain adapted to the new host that may have properties distinct from those of the original prion isolate [3].

A lack of convenient animal models capable of supporting prion infection from a range of species leads to challenges in investigating prion disease pathogenicity, clinical phenotypes and transmission barriers to infection. The European bank vole (*Myodes glareolus*) is one well-established model for prion transmission, as it lacks a significant species barrier to a wide range of prion strains from multiple species. Interestingly, four native North American vole species were recently shown to be susceptible to infection with chronic wasting disease, or CWD [4]. These included two members of the North American rodent genus *Peromyscus*, also known as deer mice. Like bank voles, these rodents are members of the taxon *Cricetidae* and are widely distributed throughout North America, from Alaska and Canada to central Mexico [5]. The prion protein primary sequences of deer mice and bank voles are nearly identical, differing by only two amino acids at positions 227 and 230. Notably, the bank vole prion protein residues E227 and S230 are key factors in regulating the conversion of normal prion proteins into their pathogenic form and play a role in influencing the “species barrier” [6,7].

Chronic wasting disease (CWD) was first identified in 1967 in captive deer in Colorado [8]. It has since spread rapidly across North America in free-ranging cervids and is endemic in the American Midwest and southern parts of Alberta and Saskatchewan in Canada. There have been no reported cases of CWD infection in humans, nor is there any direct evidence to suggest that CWD may be transmitted to humans. Animal studies show transmission of CWD to a non-human primate, squirrel monkeys, although it has not been transmitted to macaques, a species more closely related to humans [9,10]. Nonetheless, the zoonotic risk of CWD is poorly understood, and both public health officials and physicians need to remain cautious. A significant gap in knowledge arises from the lack of a model for human CWD that would enable characterization of the disease phenotype and the development and validation of specific tests. Transgenic mouse models have greatly advanced prion disease research. However, a limitation is that no single transgenic mouse model is permissive to both cervid CWD and human sCJD directly; two separate models are thus required to study each variant, which complicates direct comparisons [11,12]. Studying the transmission of sCJD alongside that of CWD in deer mice could provide valuable insights into the role of the E227 and S230 residues. This model may also serve as a tool for identifying new prion strains that could emerge if CWD were to spill over to humans.

Here, we show that deer mice are susceptible to disease following inoculation with brain homogenate from both elk with CWD and humans diagnosed with sCJD. We describe a thorough characterization of both primary transmission and the subsequent adaptations of each strain in deer mice. We show that CWD and sCJD exhibit distinct pathology and biochemical characteristics of PrP^Sc^, both on primary transmission and on passaging. Furthermore, we show that, as previously described in bank voles, sCJD of the M1 strain transmits efficiently, while deer mice are resistant to the sCJD V2 strain.

## 2. Methods

### 2.1. Brain Inocula

All procedures were performed under anesthesia, with measures taken to ensure the animals’ well-being. All infectious work was performed under conditions of biosafety level 2+ (BSL-2+) or higher. Human tissue samples (frontal cortex) were collected at autopsy of patients with Creutzfeldt–Jakob disease with the appropriate consent for use in research (Health Canada-Public Health Agency of Canada Research Ethics Board reference numbers REB 2009-036P and REB 2017-009P). Diagnosis was performed antemortem by EP-QuIC analysis of cerebrospinal fluid and confirmed by histological analysis of brain tissue and western blotting [13,14].

Obex tissue from a farmed elk with the genotype MM at codon 132 that had been confirmed to be suffering from CWD was used to generate the inoculum [15]. The animal had shown clinical signs typical of CWD in North American cervids, including weight loss, uneven hair coat, excessive salivation, aggression and ataxia. On histopathological analysis, the obex and retropharyngeal lymph node tissue showed extensive PrP^Sc^ deposition [16].

### 2.2. Animal Procedures

The animal experiments described in this study were carried out at the National Microbiology Laboratory (NML) at the Public Health Agency of Canada under an approved animal use document AUD#H23-005 (formerly H19-005) in accordance with the guidelines provided by the Canadian Council on Animal Care. Deer mice (*Peromyscus maniculatus*) used in this study were supplied by a breeding colony housed at the University of Manitoba in a pathogen-free facility. All the deer mice were acclimated for a minimum of one week before the initiation of experimental procedures. Deer mice were supplied with food and water ad libitum, provided environmental enrichment throughout the experiment, and monitored daily. Intracerebral inoculations were performed on 4- to 6-week-old male or female deer mice. For the first passage, a 10% (*w*/*v*) human or elk inoculum in phosphate-buffered saline (PBS) was prepared from the frontal cortex of human cases and the obex of the elk. A volume of 20 µL was injected into the left cerebral hemisphere under anesthesia using a Tri-Dek Stepper needle (26 × 5/32’—4.0 mm gauge). Similar 10% inocula were prepared for the second passage from the positive deer-mouse brains. Clinical scoring and behavioral analysis of inoculated deer mice were conducted bi-weekly until signs of clinical disease appeared, after which point observations were made more frequently. The clinical signs scored were hunched posture, muscle tremors, lethargy/social isolation, rapid breathing, ruffled coat (lack of grooming), aggression/excitability, circling, piloerection, ataxia, nose bulge (pain indicator) and an abolished righting reflex. Animals were euthanized once a humane endpoint was reached via perfusion under deep isoflurane anesthesia. Brains were bisected with a sagittal cut, and half the brain was fixed in formalin, whilst the other half was flash-frozen in methanol-activated dry ice and stored at −80 °C.

### 2.3. Genotyping of Deer Mice

Genomic DNA was isolated from mouse brain tissue using a phenol–chloroform extraction. Briefly, brain tissue was emulsified and incubated at 60 °C overnight in a solution of Proteinase K and ATL tissue lysis buffer (Qiagen). RNase A (Qiagen) was added the following day. Samples were extracted three times with phenol, then three times with chloroform: isoamyl alcohol. DNA was precipitated with sodium acetate (3 mM) and 100% ethanol, then dried and reconstituted in Tris-EDTA buffer. Forward primer 5′-ATGGCAACCTTGGCTACTGG-3′ and reverse primer 5′-CCAGCCTAGACCACAAGAACG-3′ were used to amplify the PRP coding region. PCR was conducted using the KAPA Hifi HotStart Ready Mix PCR kit (Roche, Basel, Switzerland) according to the manufacturer’s instructions. PCR conditions were as follows: initial denaturation at 95 °C for 3 min (1 cycle), denaturation at 98 °C for 20 s (30 cycles), annealing at 65 °C for 15 s (30 cycles), extension at 72 °C for 30 s (30 cycles) and final extension at 72 °C for 1 min/kb (1 cycle). PCR products were gel-purified using the MiniElute Gel Extraction Kit (Qiagen, Hilden, Germany) as per the manufacturer’s protocol. The DNA Core Facility of the National Microbiology Laboratory performed sequencing using an ABI 3730 DNA Analyzer.

### 2.4. Profiling and Immunochemistry of Histopathological Lesions 

Brains were formalin-fixed (10%) for approximately 48 h, then placed in the Leica Pearl (Leica) for processing. Tissues were embedded in paraffin and sectioned at 5 microns. Tissue was floated on a water bath, placed on positively charged glass slides (Leica), and left to dry at 37 °C overnight. The slides were deparaffinized and hydrated using xylenes and a decreasing ethanol wash series. Slides were placed in 98% formic acid and rinsed in water for 3–5 min washes. Hematoxylin and eosin (H&E) staining was performed to resolve and score the extent brain vacuolation, as previously described [17]. Sections were used for scoring at least three mice per challenge group. A score on a scale of 0–5 was assigned based on vacuolation in nine different brain regions: (1) medulla, (2) cerebellar cortex, (3) midbrain (superior colliculus), (4) hypothalamus, (5) thalamus, (6) hippocampus, (7) septum, (8) cerebral cortex and (9) forebrain cerebral Cortex. Scoring was performed blindly by at least two individuals, and the consensus was reported.

Immunohistochemistry was performed following antigen retrieval in 10 mM sodium citrate, pH6.0. For PrP^Sc^ staining, the anti-prion antibody 9A2 (dilution 1 in 500) was used with the Mouse-on-Mouse Elite^®^ Immunodetection Kit (Vector Laboratories, Newark, CA, USA) according to the manufacturer’s instructions. The peroxidase substrate solution for this method was prepared using the NovaRED Kit (Vector Laboratories, Newark, CA, USA). Staining for Iba1, a marker of microglia, was performed using the Polink-2 HRP Plus Rabbit DAB Detection System (OriGene, Rockville, MD, USA) and Anti-Iba1 Rabbit Antibody (Wako, Richmond, VA, USA) at a dilution of 1 in 2000 using a Decloaking Chamber (BioCare, Taoyuan, Taiwan).

### 2.5. Western Blotting

All tissues were homogenized at 10% (*w*/*v*) using single-use homogenizers (OMNI International, Kennesaw, GA, USA) according to the manufacturer’s instructions in homogenization buffer (100 mM NaCl, 10 mM EDTA, 0.5% NP-40, 0.5% sodium deoxycholate, 100 mM Tris-HCl, pH 8.0). Next, 10% brain homogenate was combined with Proteinase K (PK) and incubated at 37 °C for 1 h. PK-digested homogenates were combined with 4X NuPAGE sample buffer (Invitrogen, Waltham, MA, USA) and 1,4-dithiothreitol (Millipore Sigma, Burlington, MA, USA), then heated at 96 °C for 5 min. Next, 10 µL of sample was loaded onto Invitrogen’s 4–12% NuPAGE Bis-Tris Gel. Following electrophoresis, the samples were transferred to nitrocellulose membranes and blocked for 1 h with 5% (*w*/*v*) dry milk in TBST (20 mM Tris-HCl, pH 7.4, 150 mM NaCl, 0.1% Tween 20). Prion proteins were detected using primary antibody Sha31 (1:8000) (Cayman Chemical, Ann Arbor, MI, USA) or 9A2 antibody (1:8000) (Wageningen) diluted in milk/TBST buffer for 1 h. A secondary antibody, polyclonal goat anti-mouse HRP (Dako Denmark, Glostrup, Denmark), was used at a final concentration of 1:2000, with a 1 h incubation. Samples were detected via chemiluminescence using SuperSignal West Femto substrate (ThermoFisher Scientific, Waltham, MA, USA) and visualized with Bio-Rad’s ChemiDoc System (647SP, no light) (Bio-Rad, Hercules, CA, USA).

### 2.6. PTA Precipitation and Capillary Electrophoresis

A 10% brain homogenate (*w*/*v*) was precipitated with sodium phosphotungstate anion (NaPTA) as previously described [18,19], with modifications [20]. PTA-precipitated homogenate was then digested with PK for 1 h at 37 °C. The capillary electrophoresis assay (JESS by ProteinSimple, San Jose, CA, USA) was performed as previously described [20]. Briefly, prepared samples were denatured with the ProteinSimple 5X Fluorescent Master Mix (final concentration of 1% SDS) at 95 °C for 5 min with agitation at 1000 rpm. The proprietary electrophoresis capillary plate (ProteinSimple) was loaded according to the manufacturer’s instructions. The primary antibody, Sha31 (1:10) or 9A2 (1:15), was probed with the anti-mouse secondary antibody. The loaded plates were centrifuged at 1000 x g for 5 min to eliminate bubbles from the wells. Plates and capillaries (12–230 kDa) were loaded into the JESS equipment, and the appropriate protocol was selected using the Compass for SW software (Version 7.0). The running time of the JESS takes approximately 3 h. Data collected by Compass for SW are presented as arbitrary chemiluminescence units vs. apparent molecular weight (MW).

### 2.7. RT-QuIC Seeding

To begin seeding, 10% (*w*/*v*) homogenate was serially diluted 10-fold in PBS enhanced with 0.1% (*w*/*v*) SDS and 1% (*w*/*v*) N2 supplement (Gibco, Grand Island, NY, USA). Reactions were prepared in a 96-well plate by combining 2 μL of diluted homogenate with 98 μL of prepared master mix for reactions with final concentrations of 1 mM EDTA, 10 nM ThT, 0.002% (*w*/*v*) SDS and 10 μg of hamster rPrP substrate (residues 23–231). The substrate was prepared in-house using recombinant expression in *Escherichia coli* and purification with histidine affinity chromatography [21]. All samples were run in quadruplicate. A 10% brain homogenate from hamster 263K and a 10% brain homogenate from the PBS (mock)-infected hamsters were included for each RT-QuIC reaction plate as positive and negative technical controls, respectively. Reaction plates were sealed with optical adhesive film and ran on FLUOstar Omega microplate readers (BMG). ThT fluorescence (450 nm excitation and 480 nm emission) was measured following 15 min cycles of double-orbital shaking for 50 h. Data analysis was conducted in Omega Data analysis software (BMG) (Version 4.01) and Prism 10 (GraphPad) (Version 10.4.1).

## 3. Results

### 3.1. The North American Deer Mouse, Peromyscus maniculatus, Is Susceptible to sCJD and CWD

**Figure 1:**
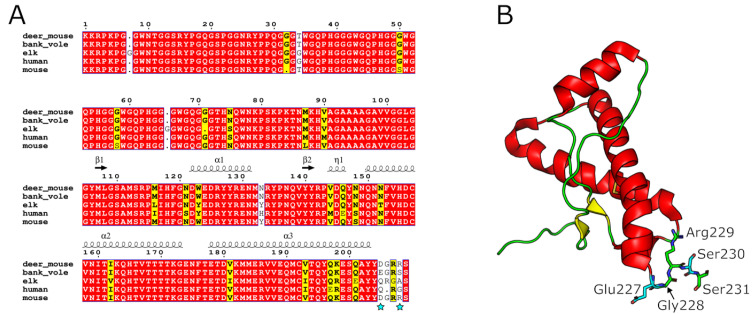
Conservation of amino acid sequence between deer mouse, bank vole, elk, human and mouse prion proteins. (**A**) Sequence alignment of deer mouse (GenBank ACV85683.1), bank vole (GenBank XP_048311321.1), elk (AAC12860.2), human (NP_000302.1) and mouse (AAH06703.1) PrP. The two residues that are not conserved between deer mouse/mouse and bank vole at the C-terminus of the PrP^C^ globular domain are indicated with cyan stars. Secondary-structure assignments are shown above the alignment based on the bank vole PrP^C^ domain, PDB entry 2K56. Sequence alignment was performed using the Muscle alignment algorithm. (**B**) Cartoon representation of the C-terminal globular domain of bank vole PrP^C^ (PDB entry 2K56). α-helices, β-strands and loop structures are depicted in red, yellow and green, respectively. The C-terminal residues 227EGRSS231 are shown as sticks, with non-conserved residues Glu227 and Ser230 highlighted in cyan.

**Figure 2:**
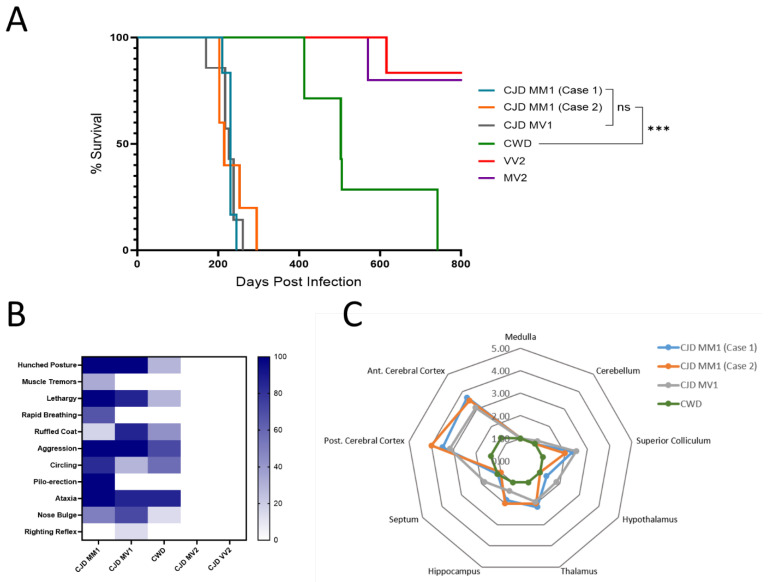
Comparison of transmission characteristics between Creutzfeldt–Jakob disease (CJD) strains and chronic wasting disease (CWD) in *Peromyscus maniculatus.* (**A**) Survival curve for *P. maniculatus* inoculated with CJD-MM1 (2 cases), -MV1, -MV2, -VV2 and CWD. Survival times for CJD-MM1 and CJD-MV1 cases were not significantly different (ns denotes no significant difference for *p* = 0.96), but both were significantly different from the CWD survival time (statistical significance is indicated by *** for *p* = 0.0003), as determined by the Mantel–Cox (log-rank) test. Mice inoculated with CJD-VV2 and CJD-MV2 did not develop clinical disease; one mouse from each of these groups died from non-disease-related causes. (**B**) Heat map showing clinical signs observed in *P. maniculatus* inoculated with CJD strains and CWD (displayed as % of inoculated mice per group). (**C**) Scoring of regional brain vacuolation for mice inoculated with CJD-MM1, CJD-MV1 and CWD-. Scoring was based on hematoxylin and eosin (H&E) staining of brain tissue, with average vacuolation scores plotted for each group.

We confirmed that all deer mice had identical *PRNP* gene sequences, with methionine at position 109, a residue important to the species barrier in a closely related species, the bank vole (Figure 1). We inoculated groups of deer mice with CWD and four different subtypes of sCJD. In sCJD, cases are classified using a combination of the electrophoretic mobility of the unglycosylated protease-resistant fragment of PrP^Sc^ as either Type 1 (19 kDa) or Type 2 (21 kDa) and are also classified using the genotype at codon 129 of the *PRNP* gene (M/M, M/V, or V/V). This results in six canonical types: MM1, MM2, MV1, MV2, VV1 and VV2 [22]. The four most common types, MM1, MV1, VV2 and MV2, were chosen for inoculation. Transmission rates were 100% for all inoculations with Type 1 sCJD and CWD, while deer mice were not susceptible to infection with the MV2 or VV2 types. Survival times are provided in Table 1. The two groups of deer mice infected with CJD subtype MM1 had mean survival periods of 229 ± 10 and 234 ± 36 days; mean survival periods for subtype MV1 and CWD were ±26 546 ± 130 days, respectively (Figure 2A).

The disease onset in sCJD-infected deer mice was characterized by behavioral changes, usually hyperactivity. Disease progression included typical signs of clinical prion disease, such as hunched posture, piloerection and circling, as well as aggression. Infection with sCJD in deer mice was unique in that it was accompanied by hyperexcitability and aggression to an extent not typical of previous models of prion disease we have studied in rodents. These clinical signs were less prominent in CWD-infected mice, in which ataxia was the most predominant clinical feature (Figure 2B).

**Figure 3:**
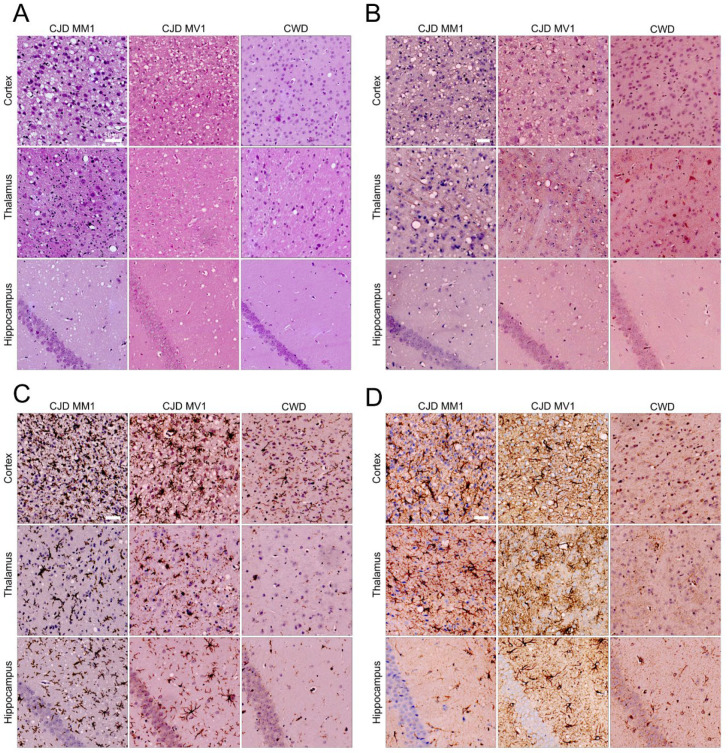
*P. maniculatus* inoculated with CJD (MM1 & MV1) and CWD exhibit prion-strain-specific neuropathology. Representative brain sections of the cortex, thalamus and hippocampus were stained with (**A**) H&E, (**B**) 9A2, (**C**) IBA1 and (**D**) GFAP. Scale bar: 50 µm (applies to all sections).

Neurodegeneration was characterized by scoring vacuolation severity in nine brain regions for each group of CJD and CWD mice, with the resulting lesion profiles shown in Figure 2C [17]. Spongiform changes were remarkably prominent in the anterior and posterior cerebral cortex in all mice infected with MM1 and MV1 CJD, with the medulla and cerebellum relatively spared. CWD-infected mice showed less extensive vacuolation overall. Immunohistochemical staining was performed using the 9A2 antibody to detect PrP^Sc^, and IBA1 and GFAP were used to detect activated microglia and astrocytes, respectively (Figure 3). Following inoculation with MM1 and MV1 CJD, PrP^Sc^ accumulation was intense and presented as fine deposits in the cortex, with more coarse deposition in the thalamus, hypothalamus and midbrain. Coarse staining was frequently intracellular and appeared surrounding vacuoles. Less deposition was apparent in the cerebellum and hippocampus. In CWD-infected animals, staining was evident in most brain regions, apart from the cerebellum. However, the deposition was more granular and was mainly intracellular, with frequent well-defined plaques that were particularly prominent in the thalamus (Figure 3B). Astrocytic gliosis and activated microglia were apparent in the MM1- and MV1-infected mice, with remarkable staining in the cortex. Staining for gliosis and activated microglia was less apparent in CWD-inoculated mice across all brain regions (Figure 3C,D).

### 3.2. Protease-Resistant PrP^Sc^ and Prion Seeding Activity Is Abundant in CJD- and CWD-Infected Deer Mice

**Figure 4:**
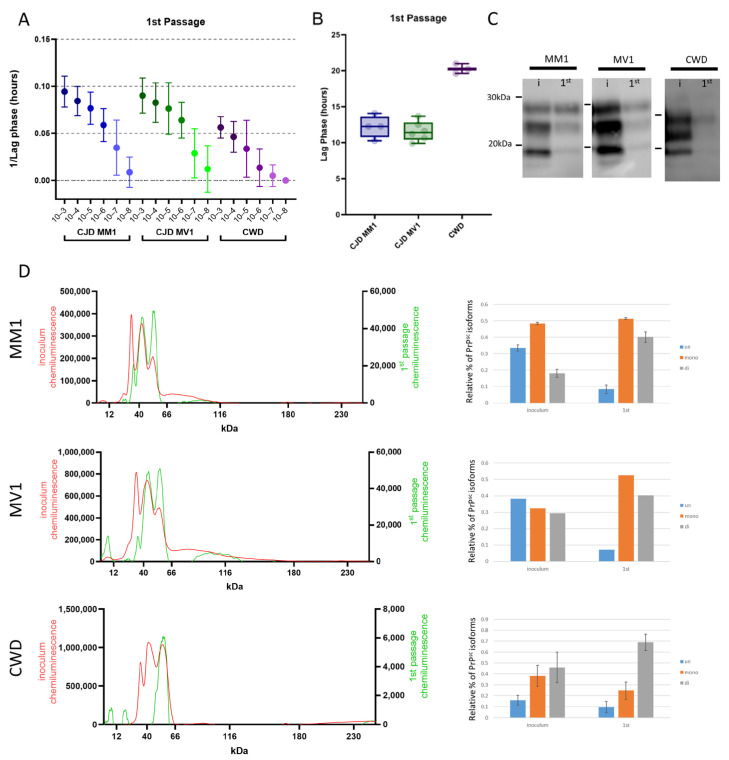
Seeding properties and biochemical subtyping of protease-resistant PrP in passaged cases of sCJD and CWD in *P. maniculatus*. (**A**) RT-QuIC results for serially diluted 10% brain homogenate from the three passaged cases represented as 1/lag phase of 10^−3^ to 10^−8^ dilutions. (**B**) To show the significant differences between the CWD and sCJD cases, the lag phase in hours for the 10^−3^ dilution of each sample is displayed. (**C**). Western blot analysis of protease-resistant PrP for the inoculum (i) and the first passage in deer mice (first) of the three cases (final PK concentration 100 µg/mL). (**D**) Capillary electrophoresis of the inoculum (in red) and the first passage (in green) shows the un-, mono- and diglycosylated forms of protease-resistant PrP for each case. The three glycoforms are quantified on the right.

All deer mice that succumbed to sCJD and CWD displayed robust seeding activity in brain tissue on assay using the real-time quaking-inducing conversion assay (RT-QuIC) (Figure 4A). When full-length hamster PrP was used as a substrate, the seeding efficiency of CWD- and sCJD-passaged deer mice was specific to each strain. CWD presented with a slower lag phase compared to sCJD. Direct comparison of the lag phase in hours between sCJD (around 12 h) and CWD (around 20 h) showed a significant difference at a 10^−3^ brain-homogenate dilution (Figure 4B). We find the 10^−3^ dilution is optimal for sample comparisons, as it is sufficiently dilute to avoid fluorescence inhibition due to sample composition and variability between replicates is minimal compared to higher dilutions.

The biochemical characteristics of human and deer mouse CJD and of elk and deer mouse CWD were studied by comparing their electrophoretic mobility and glycoform patterns after protease digestion using western blot and capillary electrophoresis. Of note, antibodies to protease-resistant PrP^Sc^ bound poorly to deer mouse protein; the antibody Sha31, previously used to detect PrP^Sc^ in bank voles, provided the best signal [23]. We found that on transmission, the glycoform profiles of the original inoculum and all three groups of infected deer mice were distinct, reflecting adaptation in the new host (Figure 4C). Following both MM1 and MV1 transmission, the diglycosylated and monoglycosylated protease-resistant fragments of PrP^Sc^ were dominant in the deer mice, with marked reduction in the levels of unglycosylated protein, whilst CWD transmission produced only a diglycosylated PrP^Sc^ fragment when compared to the original inoculum. Due to the weak signal observed in the CWD-infected mouse sample, we performed a PK titration assay, which revealed that PrP^Sc^ in this sample is sensitive to PK digestion. The strongest PrP^Sc^ signal was detected at 5 µg/mL, with signal intensity progressively diminishing as the PK concentration increased (Supplementary Figure S1). Capillary electrophoresis was used to provide further clarity, as it allows for effective quantification of each glycoform (Figure 4D). Transmission of sCJD in deer mice resulted in an approximate four-fold reduction in unglycosylated PrP^Sc^ and more diglycosylated isoforms when they were visualized as a percentage of total protease-resistant PrP^Sc^. The CWD model differs in that the diglycosylated form is predominant in the passaged group compared to the initial inoculum. However, the glycoform profile shows changes that are less marked than those that were seen for sCJD (Figure 3D).

### 3.3. Adaptation of sCJD MM1 and CWD on Second Passage

**Figure 5:**
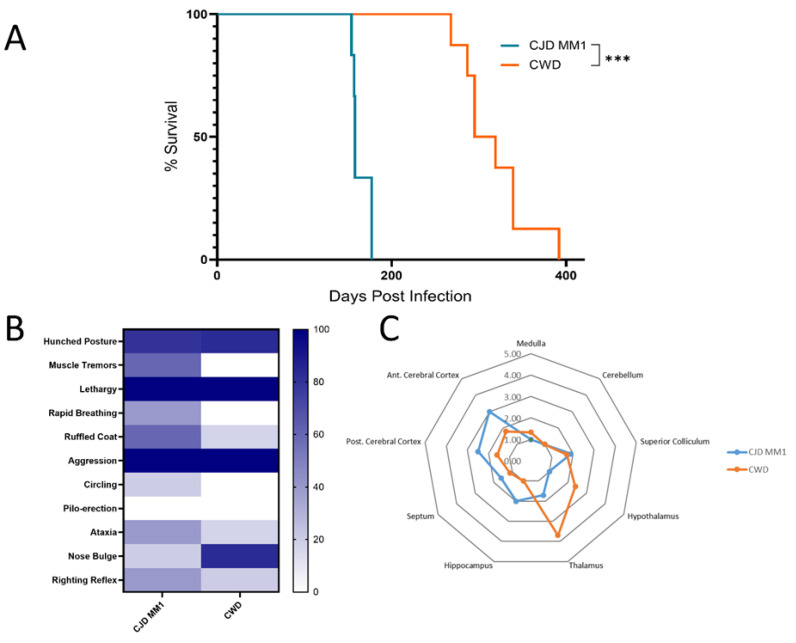
Transmission characteristics of CJD-MM1 and CWD following passaging in *P. maniculatus*. (**A**) Survival curve for *P. maniculatus* upon second passaged inoculation with CJD-MM1 and CWD. Survival times for CJD-MM1 and CWD were significantly different (statistical significance is indicated by *** for *p* = 0.0001), as determined by the Mantel–Cox (log-rank) test. (**B**) Heat map showing clinical signs observed in *P. maniculatus* inoculated with passaged CJD-MM1 and CWD. (**C**) Scoring of regional brain vacuolation for passaged CJD-MM1 and CWD-inoculated mice. Scoring was based on hematoxylin and eosin (H&E) staining of brain tissue, with average vacuolation scores plotted for each group.

We performed a second passage of sCJD MM1 and CWD in deer mice to study adaptation of prion strains more closely. Given that in humans, sCJD MM1 and MV1 cases are phenotypically indistinguishable and that characterization suggests the prions are identical biochemically, we chose a single brain homogenate with the M1 strain as the inoculum. The extent of adaptation in a new host can be inferred by comparing the decrease in survival time between the primary transmission and further passages [3,24]. We determined that survival periods decreased after the first passage for both groups, with the MM1-inoculated group averaging survival times of 164 ± 10 dpi and the CWD-inoculated group averaging 317 ± 34 dpi (Figure 5A). These results represent 28.4% and 41.9% reductions in survival period for the M1- and CWD-inoculated groups, respectively, compared with the first passage (Table 1). Furthermore, changes were also evident in the clinical signs shown by each group of infected mice on second passage. This was more marked in the CWD-inoculated group, in which the deer mice primarily exhibited hunched postures, lethargy and aggression, in contrast to the ataxia and circling predominant during the first passage (Figure 5B).

**Figure 6:**
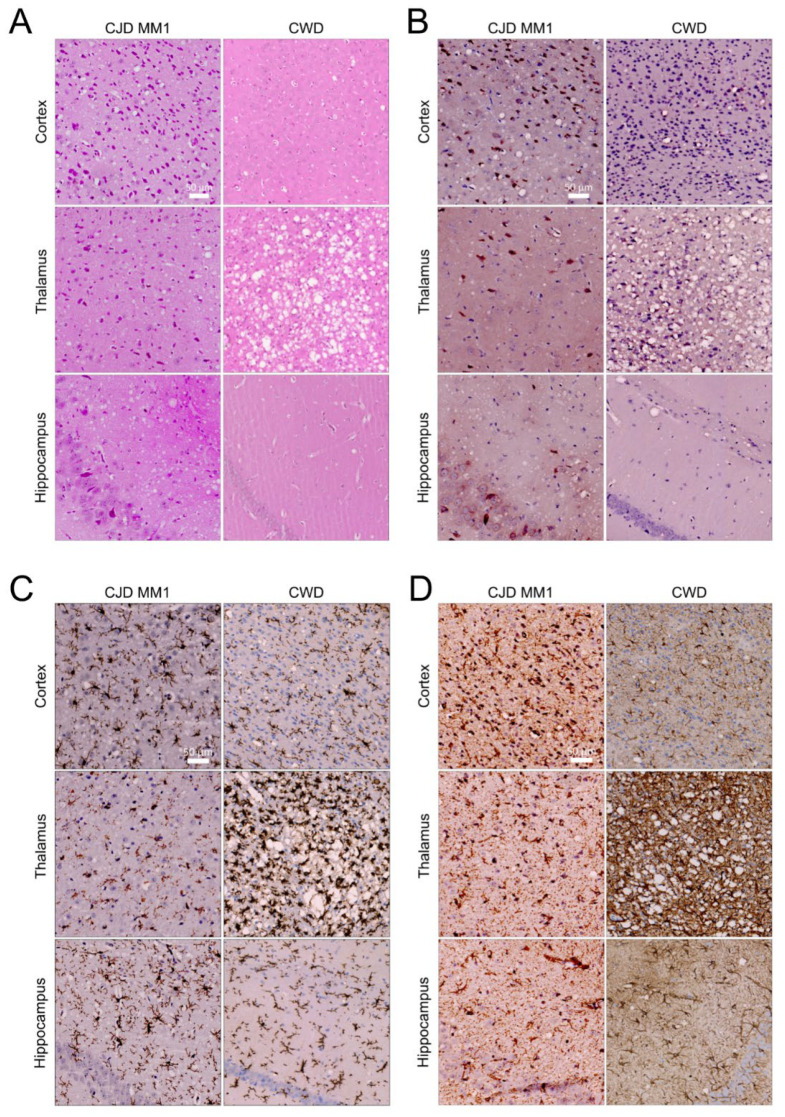
Passaging of CJD-MM1 and CWD in *P. maniculatus* leads to neuropathological alterations indicative of strain adaptation. Representative brain sections of the cortex, thalamus and hippocampus were stained with (**A**) H&E, (**B**) 9A2, (**C**) IBA1 and (**D**) GFAP. Scale bar: 50 µm (applies to all sections).

Vacuolation profiles of the sCJD M1-inoculated deer mice remained relatively constant upon passaging, albeit with slightly milder vacuolation in the cortex. Conversely, the vacuolation distribution and intensity for the CWD-inoculated group significantly differed from that seen in the first passage. In the second passage, mice inoculated with CWD exhibited remarkable vacuolation in the thalamus and moderate vacuolation in both the hypothalamus and midbrain (Figure 5C). Furthermore, there was intense staining for Iba1 (activated microglia) and GFAP (astrocytic gliosis) in the thalamus (Figure 6).

**Figure 7:**
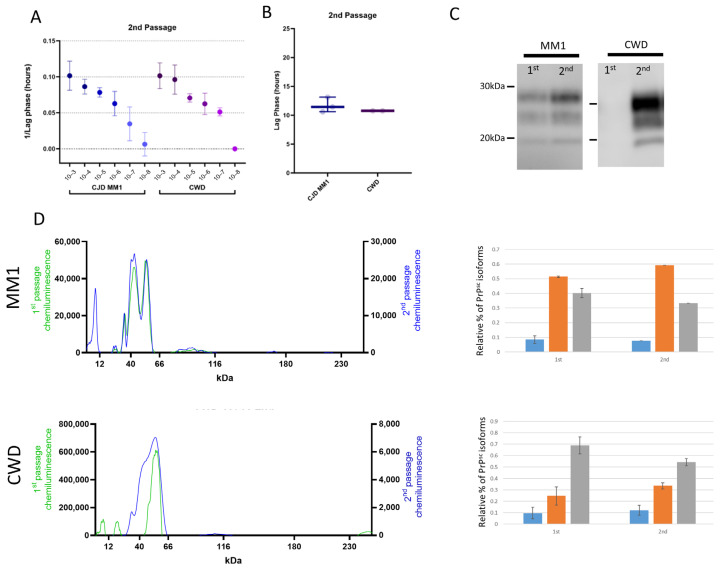
Seeding properties and biochemical subtyping of protease-resistant PrP in second-passaged cases of sCJD and CWD in P. maniculatus. (**A**) RT-QuIC results of serially diluted 10% brain homogenate from the two passaged cases represented as 1/lag phase of 10–3 to 10–8 dilutions. (**B**) The difference between CWD and sCJD is less apparent in the second passage, as represented by the lag phase in hours for the 10–3 dilution of each sample. (**C**) Western blot analysis of protease-resistant PrP for the first and second passages in deer mice. (**D**) Capillary electrophoresis of the first passage (in green) and the second passage (in blue) was used to characterize the un-, mono- and diglycosylated forms of protease-resistant PrP, which are quantified on the right.

Seeding efficiency was more similar between sCJD M1 and CWD in the second passage. All dilutions had similar lag phases, and at a dilution of 10^−3^, lag phases were almost identical between the two groups (~11 h) (Figure 7A,B). The electrophoretic mobility of protease-resistant PrP^Sc^ from brains of sCJD M1 deer mice was almost identical between first and second passages (Figure 7C,D). The monoglycosylated isoform was the predominant variety, whereas the unglycosylated isoform was the most minor. CWD maintained its unique glycoform profile; however, the dominance of the diglycosylated band was less marked than it had been in the first passage (Figure 7C,D).

**Figure 8:**
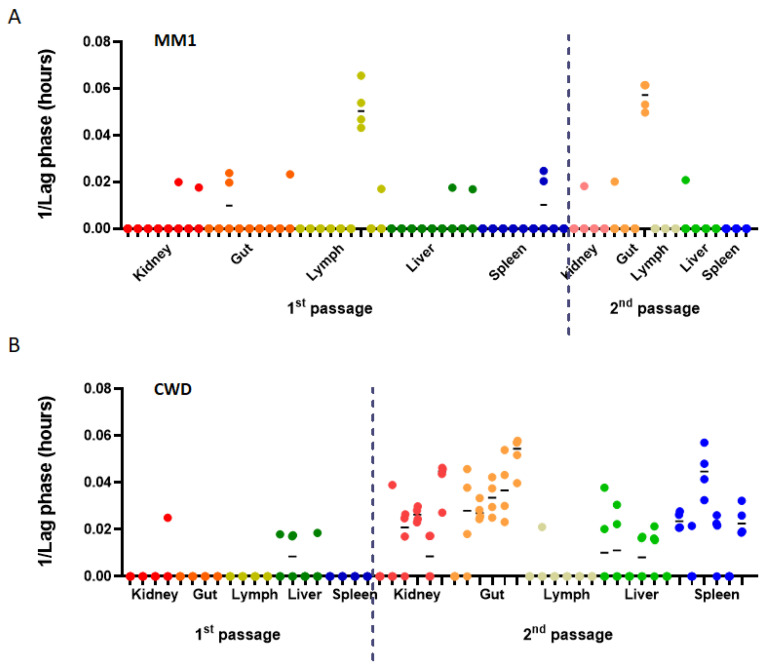
Seeding properties of peripheral tissue between the first and second passage in deer mice of sCJD MM1 and CWD. 10% homogenates of kidney (red), gut (orange), lymph nodes (yellow), liver (green) and spleen (blue) of multiple mice in the first passage (left) and second passage (right) for both sCJD MM1 (**A**) and CWD (**B**) were subjected to RT-QuIC. The figures show 1/lag phase in hours.

We measured prion seeding in extraneural tissues of deer mice infected with sCJD MM1 and CWD to determine whether there were further phenotypic differences between the two strains. RT-QuIC was performed on 10% homogenates of kidney, gut, lymph nodes, liver and spleen of mice from the first and second passages of both sCJD MM1 and CWD in mouse models. A lack of consistent seeding was observed in the organs of all mice inoculated with sCJD MM1. This pattern was similar between first and second passages (Figure 8A). Spontaneous seeding was not detected in control tissues from uninfected animals, except in the gut, where occasional random seeding events were observed (Supplemental Figure S2). On first passage of CWD, a similar lack of seeding in extraneural tissues was observed; however, CWD seeding activity increased to levels that were significant for all tissues tested in the second passage (Figure 8B). We analyzed these tissues by immunoblot and capillary electrophoresis but did not detect any PrP^Sc^ bands. We concluded that a combination of the low concentration of prion protein in all these tissues and the poor affinity shown by antibodies to deer mouse PrP^Sc^, which was particularly evident for CWD, led to the protein being undetectable. We conducted further tests on feces from infected animals to assess whether prions were being shed into the environment while the animals were symptomatic. Our findings revealed no seeding activity in the feces of rodents infected with sCJD or CWD.

## 4. Discussion

In this study, we show that the North American deer mouse, *Peromyscus maniculatus*, is permissive to infection with both human and cervid prion diseases. Deer mice, like the well-characterized prion rodent model, bank voles (*Myodes glareolus*), appear to be highly susceptible to prions from many species and differ by only two amino acids in their PRNP gene product [25,26,27,28,29]. Given that their range spans North America and that they are sympatric with the cervids experiencing the spreading CWD epidemic, understanding the potential for CWD susceptibility amongst these wild rodents is important. The species barrier appears high, as evidenced by long incubation periods (averaging 595 days) for CWD in deer mice that were reported in a previous study of CWD transmission in white-tailed deer [4]; similarly, an incubation period of 546 ± 130 days was reported in this study, though reinfection could lead to adaptation. It is known that CWD strain characteristics may evolve over time, potentially altering the transmissibility and host range. There are two main strains of CWD, CWD1 and CWD2 [30]. While they differ in their clinical and neuropathological profiles, they cannot be distinguished by biochemical properties. CWD1 typically has shorter incubation periods in rodent models. Although the strain of the elk isolate in this study is unknown, it is possible CWD2 was used, which could result in longer incubation times. However, in bank voles, CWD2 has a mean incubation period of 226 dpi, which is still much shorter than that in deer mice, highlighting the strong species barrier to CWD in deer mice regardless of strain [4,28].

The ability to transmit human sCJD and CWD in the same host enabled us to assess the adaptation characteristics of each strain. We found that although survival times were shortened by 30–40%, indicating significant adaptation, on second passage, sCJD and CWD maintained unique strain characteristics, including distinct glycoforms and neuropathological profiles. Given the maintenance of biochemical strain characteristics on passaging, it seems reasonable to conclude that the significant species barrier to transmission of CWD to humans would be maintained following reverse transmission from deer mice to cervids or further interspecies transmission. However, additional experimental work on interspecies transmission and the environment is required to extrapolate these results to real-world scenarios. Techniques such as protein misfolding cyclic amplification (PMCA) using human substrates could provide further data to determine whether transmission of CWD to deer mice lowers the barrier to transmission to humans.

A further striking and distinctive feature of deer mouse CWD was the finding that seeding activity was widely disseminated in extraneural tissues following adaptation in all mice assessed. Similar adaptation was not apparent in the sCJD-infected mice, except in a single animal that showed seeding in the gut. The levels of PrP^Sc^ were likely to be low in these tissues. Further confounding immunoblot detection were the low affinities we found for all available antibodies for detection of deer mouse PrP^Sc^. This made western blotting and capillary electrophoresis techniques challenging for semi-quantitative analysis or detection of low protein levels. Although immunoblot techniques were not sensitive enough to confirm the presence of PrP^Sc^ in these tissues, the sensitivity of detection of infectious prions was equivalent between deer mouse CWD and sCJD in the RT-QuIC seeding assay. We saw that the lag phase was similar on second passage, and detection of each strain was possible down to a 10^−7^ dilution of brain homogenate, indicating that seeding activity was similar for both strains. We concluded that the seeding observed in extraneural tissues was specific to CWD adaptation and was either due to increased overall PrP^Sc^ propagation following CWD passaging or due to strain adaptation leading to the expansion of PrP^Sc^ deposition in extraneural tissues.

Given the similarity between the sequence of PRNP in deer mice and the sequence of PRNP in the widely used bank vole model, we expected to observe transmission-barrier effects similar to those previously reported [28,31]. However, incubation periods for the primary transmission of MM1 and MV1 sCJD (M1 strain) were approximately 20% longer than in bank voles, whilst CWD incubation was extended even further and was ~50% longer in deer mice. We found that as in bank voles, sCJD from cases with genotypes MV2 and VV2 did not transmit to deer mice, indicating that the presence of the D227/R230 residues did not influence cross-species transmission of the V2 strain. The transmission of the sCJD VV2 and MV2 strains is linked to a common strain, referred to as V2 [2]. When V2 variants are transmitted to a range of animal models (such as non-human primates, transgenics, bank voles etc.), they typically exhibit inefficient attack rates and/or long incubation periods relative to the M1 sCJD strain [25,31,32,33]. In contrast, in Tg HuVV mice, the sCJD V2 strains exhibit enhanced transmission efficiency [34]. This discrepancy suggests that the V2 strain is less virulent than the M1 strain in animal models lacking the codon 129V allele, a conclusion further supported by the findings in the deer mouse model. The resistance of deer mice to infection by sCJD V2 types may be considered a limitation of this model for detecting specific atypical human cases of sCJD. However, the finding that deer mice were more permissive to transmission of CJD than CWD was interesting. Both species share the asparagine residues at positions 155 and 170 of bank vole PrP^C^, and these have been shown to be significant factors in its competency to replicate multiple prion strains [27,35]. The primary sequence of deer mouse PrP^C^ differs from that of bank vole PrP^C^ by only two amino acids at the C-terminus, D227 and R230, suggesting critical roles for these residues in cross-species transmission, especially in the case of CWD.

The extreme C-terminus of the prion protein has been described as a linchpin in the control of the conversion of PrP^C^ to PrP^Sc^, with the bank vole residues E227 and S230 functioning as the primary residues promoting efficient templated misfolding [6,7,36]. However, further studies using an in vitro conversion system and specifically targeting the bank vole residues at positions 227 and 230 have yielded conflicting results. Work by the same group suggests that the presence of E227 and S230 in the primary sequence of wild-type bank vole reduces the susceptibility to cross-species prion infection. In a cell culture model of prion infection, replacement of bank vole residues E227/S230 with mouse D227/R230 rendered cells more permissive for replication of various hamster and mouse prion scrapie strains. This was determined by the finding that these cells exhibited much higher levels of PrP^Sc^ than did cells expressing wild-type bank vole PrP on challenge [37]. Infection of deer mice provides an in vivo model in which to test this hypothesis further, specifically, to determine whether this translates to a reduction in incubation times compared to those seen in bank voles. Our finding in this work does not support the hypothesis that substitution of the bank vole residues E227/S230 with the mouse residues D227/R230 universally results in increased permissivity to prions. It seems that the contribution of residues 227 and 230 to the species barrier is strain-dependent. One further explanation is that the higher levels of PrP^Sc^ observed by Arshad et al. in cell lines expressing the mouse D227/R230 residues reflects a low barrier for the initial seeding event but increased stability and accumulation of the refolded protein.

This study establishes the North American deer mouse (*P. maniculatus)* as a novel and valuable model for investigating CJD and CWD transmission and adaptation. Notably, adaptation of CWD in mice resulted in widespread extraneuronal seeding and underscores the utility of the deer mouse model in the investigation of prion strain dynamics.

## Figures and Tables

**Table 1:** Survival times of *P. maniculatus* infected with sCJD and CWD.

	First Passage		Second Passage		% Decrease in Survival Time Upon Passaging
Inoculum	Survival Time (days ± SD)	Clinical Disease	Survival Time (Days ± SD)	Clinical Disease	
MM1 (Group 1)	229 ± 10	6/6	164 ± 10	6/6	−28.4%
MM1 (Group 2)	234 ± 36	5/5			
MV1	224 ± 26	7/7			
MV2	>600 *	0/7			
VV2	>1000 *	0/7			
CWD	546 ± 130	7/7	317 ± 34	8/8	−41.9%

* A single mouse from both the MV2 and VV2 groups died from causes not related to prion disease. The time of death for these mice has been excluded from the survival times presented in this table.

## Data Availability

All data generated during this study are included in the manuscript.

## Supplementary Materials

The following supporting information can be downloaded at: https://www.mdpi.com/article/10.3390/v17040576/s1, Figure S1: PK-titration series of the original CWD inoculum and deer mouse-passaged CWD brain homogenate.; Figure S2: Seeding properties of peripheral tissue in uninfected control deer mice.; Figure S3: Heat map showing the seeding efficacy of the original inoculum for human and elk isolates across a dilution series.
