# Supplementary material for: Transmission and Characterization of Creutzfeldt–Jakob Disease and Chronic Wasting Disease in the North American Deer Mouse
Source: Viruses. 2025 Apr 16;17(4):576. doi: 10.3390/v17040576 (PMC12031182; doi:10.3390/v17040576)
Supplement: Supplementary file 1 [file viruses-17-00576-s001.zip › viruses-3518193-supplementary.pdf]

Supplementary Materials

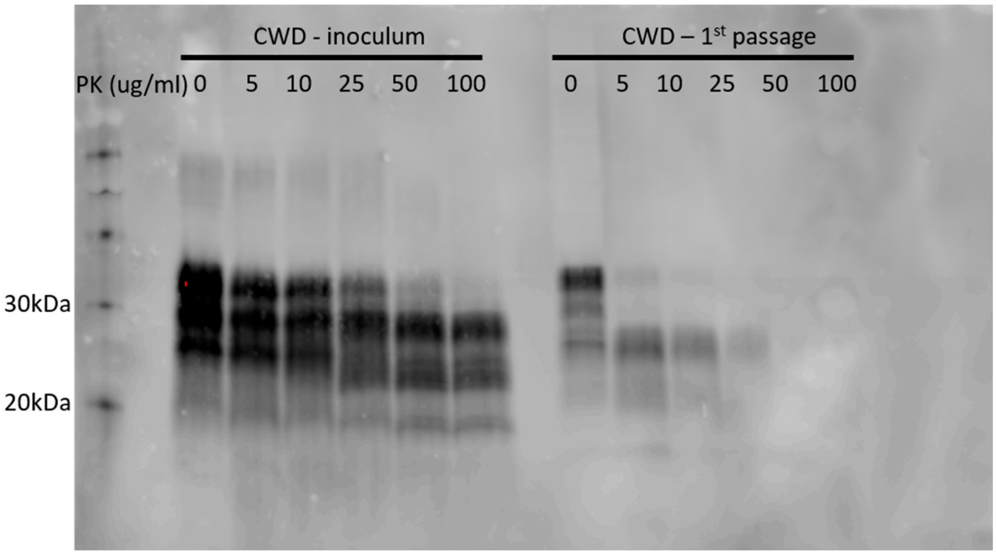

Figure S1: PK-titration series of the original CWD inoculum and deer mouse-passaged CWD brain homogenate. For this assay, 10% brain homogenates from both the original CWD-positive elk and the CWD-passaged deer mice were digested with varying concentrations of PK (5 µg/mL, 10 µg/mL, 25 µg/mL, 50 µg/mL and 100 µg/mL). Samples were probed with the primary antibody Sha31 (1:8000).

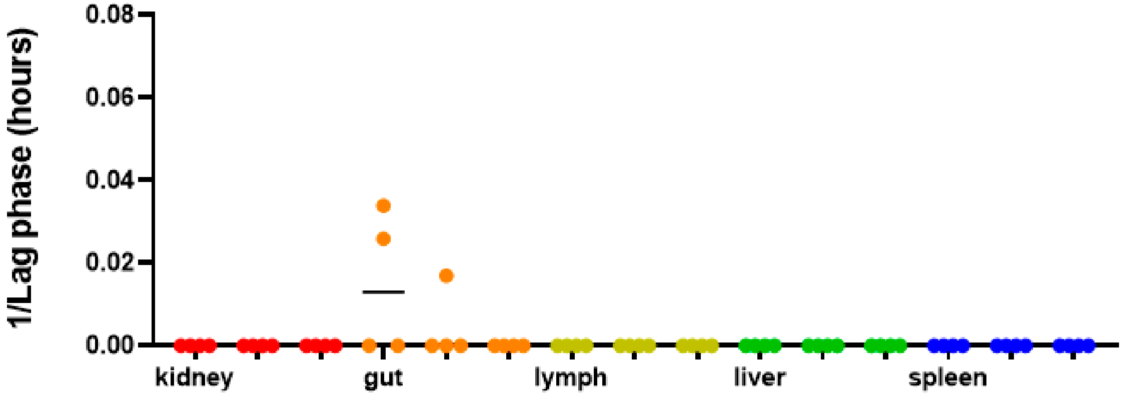

Figure S2. Seeding properties of peripheral tissue in uninfected control deer mice. For this assay, 10% homogenates of kidney (red), gut (orange), lymph nodes (yellow), liver (green) and spleen (blue) of multiple mice were subjected to RT-QuIC. The figures show 1/lag phase in hours.

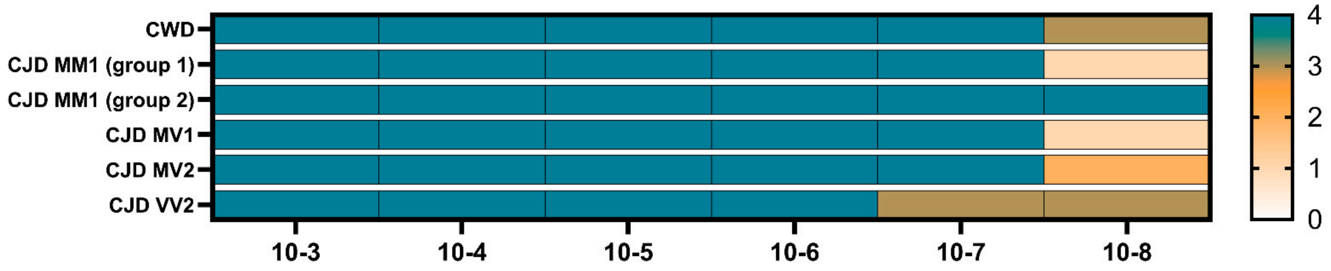

Figure S3. Heat map showing the seeding efficacy of the original inoculum for human and elk isolates across a dilution series. A 10% brain homogenate was serially diluted up to 10^{-8}, with dilutions 10^{-3} to 10^{-8} analyzed in quadruplicate using RT-QuIC. The heat map depicts the number of positive replicates (total of four replicates per sample) observed for each dilution of the original inoculum in RT-QuIC. All inocula exhibited similar seeding characteristics.
